# Transcriptomics analysis for the identification of potential age-related genes and cells associated with three major urogenital cancers

**DOI:** 10.1038/s41598-020-80065-y

**Published:** 2021-01-12

**Authors:** Jinlong Cao, Jianpeng Li, Xin Yang, Pan Li, Zhiqiang Yao, Dali Han, Lijun Ying, Lijie Wang, Junqiang Tian

**Affiliations:** 1 Department of Urology, The Second Hospital of Lanzhou University, Lanzhou, 730000 People’s Republic of China; 2 Key Laboratory of Urological Diseases of Gansu Provincial, Lanzhou, 730000 People’s Republic of China; 3 Reproductive Medicine Center, The Second Hospital of Lanzhou University, Lanzhou, 730000 People’s Republic of China; 4 Department of Gynecology, The Second Hospital of Lanzhou University, Lanzhou, 730000 People’s Republic of China

**Keywords:** Cancer, Computational biology and bioinformatics, Biomarkers, Oncology, Urology

## Abstract

Age is one of the most important risk factors of the occurrence for tumor patients. The majority of patients with urogenital cancers are the elderly, whose clinical characteristics are greatly affected by age and ageing. Our study aimed to explore age-related genes, cells, and biological changes in three common urogenital cancers via integrative bioinformatics analysis. First, mRNA (count format) and clinical data for bladder cancer, prostate cancer and renal cell carcinoma were downloaded from the Cancer Genome Atlas (TCGA). Through the comparison of clinicopathological characteristics, genes expression and cells infiltration between the old group and the young group, it was found that the clinical characteristics, genes and cells in the tumor microenvironment of different ages were quite different. And 4 key cells, 14 hub genes and some potential pathways were identified and considered as important factors. More importantly, we analyzed the differential landscape of the genes and cells from different perspectives, and confirmed its importance. In conclusion, we identified genes and cell types associated with age-related changes in the tumour microenvironment in urogenital cancer patients. These genes and cell types may play a critical role in the age-associated differences in clinicopathological characteristics among urogenital cancers, thus providing a link between ageing and cancer occurrence. The findings of this study may pave the way for the development of age-tailored approaches to treat cancer and other age-related diseases.

## Introduction

Age is one of the most important risk factors for cancer, and the occurrence and prognosis of cancer patients is highly influenced by the age at diagnosis^1^. Although people of all ages can develop tumours, the risk of most malignancies increases drastically with ageing. The average life expectancy has increased significantly over the past few decades due to the progress of modern medicine, and the incidence of almost all cancer types is increasing year by year^2^. The biological and genomic characteristics of cancers can differ immensely depending on patient age and tumour type, leading to differences in clinical characteristics and treatment outcomes^3^. Although the relationship between ageing and cancer is now evident, the mechanisms underlying the effects of ageing on tumour occurrence and progression remain elusive.

It is well known that the occurrence of tumours is a result of the accumulation of genetic mutations, and a process from quantitative to qualitative change. The accumulation of mutations and cellular damage over time contributes to the higher incidence of cancer seen in the elderly^4^. Importantly, epidemiological studies have shown that half of all tumours occur in people aged over 70 years^5^. Additionally, elderly cancer patients typically have a poor prognosis. Most young adult cancer patients have a better prognosis and survival rates than older cancer patients, although some young individuals with cholangiocarcinoma, breast cancer, and cervical cancer, among other tumour types, have a poor prognosis^6–8^. Although most tumour types are more common in the elderly, leukaemia, retinoblastoma, and nephroblastoma are more frequent in children^9^. Additionally, the average age at cancer occurrence has decreased significantly for certain tumour types, including colorectal and lung cancers. For these tumours, age is negatively correlated with malignancy^10,11^. Nonetheless, older patients are less responsive to immunotherapy than younger patients^12^. Mounting evidence also suggests that patients with the same tumour type can have different clinical characteristics depending on their age. Better understanding of the relationship between age and tumour occurrence will enable us to develop more effective, personalised cancer therapies and thus improve patient outcomes.

Recent advances in ageing and immunosenescence^13,14^ have deepened our understanding of age-related changes in the tumour microenvironment (TME). Ageing and cancer are tightly interconnected, and all TME components are influenced by ageing, which can contribute to tumour occurrence^15^. Gene mutations and cell infiltrates in the TME may differ by age, which could explain the differences in clinicopathological characteristics among patients in different age groups. Understanding the role of ageing in the TME, and by extension cancer development and progression, may facilitate the development of novel diagnostic and therapeutic approaches tailored according to patient age^16^.

Urogenital cancers account for approximately 14% of all human cancers in industrialised countries^17^. Kidney, prostate, and bladder tumours are the most common urogenital cancer types and are among the 10 most prevalent cancers in men^2,18^. Urogenital cancers are more frequent in elderly patients, and age has a clear effect on the treatment outcome in these cases. In this study, we employed various bioinformatic tools to investigate the influence of age on the prognosis of urogenital cancers. We also analysed potential age-related genes and cells in the TME. We identified 14 hub genes and 4 key cell types affecting the genomic and clinical characteristics of urogenital cancers in accordance with ageing. The findings of this study could facilitate efforts to determine the effects of ageing on other cancers and diseases.

## Materials and methods

### Data sources and pre-processing

Urogenital cancers are more frequent in elderly patients, and kidney, prostate, bladder tumours are the most common urogenital cancers. The RNA-seq expression profiles (count format) and clinical data of kidney, bladder, and prostate cancers were obtained from the Cancer Genome Atlas (TCGA) using the GDC-client tool. We used data from these three tumours to demonstrate shared characteristics of aging in the TME across urogenital cancers. All statistical analyses were performed using R version 4.0.0 software (R Foundation for Statistical Computing, Vienna, Austria) and GraphPad Prism software (version 8.0; GraphPad Software, La Jolla, CA, USA). After excluding cases with missing age and survival data (4 bladder cancer cases and 3 renal cell carcinoma cases), the patients were divided into young and old groups based on median age. The number of cases included median ages were as follows: bladder cancer, 405 cases (median age, 69 years), prostate cancer, 500 cases (median age, 61 years), and renal cell carcinoma, 884 cases (median age, 60 years).

### Influence of age on clinical characteristics of urogenital cancers

Kaplan–Meier analyses were performed to assess the overall survival of patients in each group and by cancer type. Survival analyses were performed using the log-rank test, performed with GraphPad Prism software. The major clinical characteristics of the patients were also assessed.

### Identification of age-related genes

Differentially expressed genes (DEGs) between the two groups were identified using edgeR package, and false discovery rate (FDR) were used for adjust to *P*-value. Genes with |log_2_ fold-change (FC)| > 1 and adjust *P-*value < 0.05 were considered as significant DEGs. Venn diagram analysis (https://bioinfogp.cnb.csic.es/tools/venny/) was performed to identify the common DEGs among the three urogenital cancer types.

### Enrichment analysis of the common DEGs

Gene Ontology (GO) and Kyoto Encyclopaedia of Genes and Genomes (KEGG)^19,20^ enrichment analyses were used to assess the structure, functions, and pathways of the common DEGs. These analyses were conducted using the clusterProfiler R package^21^; count ≥ 3 and *P* < 0.05 were used as enrichment cut-offs. The top 10 biological processes, molecular functions, cellular components, and KEGG pathways were identified. The clueGO Cytoscape plugin was used to further analyse biological processes and KEGG pathways.

### Protein–protein interaction (PPI) network analysis and hub gene identification

The 188 DEGs were imported into the STRING database (https://string-db.org/), and PPI analysis was performed using a combined score of ≥ 0.4. There were 139 nodes and 317 edges in the PPI network. The network was reconstructed using Cytoscape, and the cytoHubba plugin was used to identify the top 30 genes in the network using five different algorithms: maximal clique centrality (MCC), density of maximum neighbourhood component (DMNC), maximum neighbourhood component (MNC), edge percolated component (EPC), and the degree method. Genes identified by all of the different algorithms were considered as hub genes.

### Relationship between hub genes and clinical characteristics

Univariate Cox regression analysis was used to assess the role of hub genes in patient prognosis, using sex and age as covariates. Receiver operating characteristic (ROC) analysis of hub genes was performed using the pROC R package to assess the differentiation of these genes between the two groups of patients. The relationship between the mRNA levels of the hub genes and patient prognosis in the three urogenital cancers was visualised using the ggplot2 R package. We used the website cBioPortal (http://www.cbioportal.org/)^22^ to identify mutations in hub genes in the following datasets: bladder cancer (TCGA, Cell 2017), kidney chromophobe (TCGA, PanCancer Atlas), kidney renal clear cell carcinoma (TCGA, PanCancer Atlas), kidney renal papillary cell carcinoma (TCGA, PanCancer Atlas), and prostate adenocarcinoma (TCGA, PanCancer Atlas). The relationship between mutations in the hub genes and clinical characteristics was assessed using the WGCNA R package.

### Co-expression analysis of hub genes

According to the “guilt by association” theory, functionally related genes are often co-expressed^23^. Hence, co-expression analysis can provide insight into the function of poorly annotated genes. The genes co-expressed with the hub genes were identified using PCViz (http://www.pathwaycommons.org/pcviz). Then, the chromosomal distribution of hub genes and co-expressed genes was determined using the RIdeogram R package^24^. Metascape (https://metascape.org/) was used for further enrichment analysis of hub genes and co-expressed genes^25^.

### Changes in cell infiltration patterns

xCell (https://xcell.ucsf.edu/) predicts the levels of 64 cell types in tissues based on gene expression data^26^. We used the limma R package for differential analysis of cell infiltrates. |log_2_ FC| > 1 and *P* < 0.05 were set as cut-offs, and the results were visualised using a volcano map. Venn diagram analysis was performed to identify cell levels in the three TMEs. Univariate Cox regression analysis was performed to assess the relationship between cell levels and patient prognosis, and the results were visualised using ggplot2. The association between cell infiltration levels and clinical characteristics in bladder cancer patients was analysed using WGCNA R package.

### The relationship between key cells and hub genes

The corrplot R package was used to perform a Pearson correlation analysis of hub genes and key cells, and thereby acquire insight into their possible biological links.

## Results

### Influence of age on the clinical characteristics of urogenital cancers

**Figure 1:**
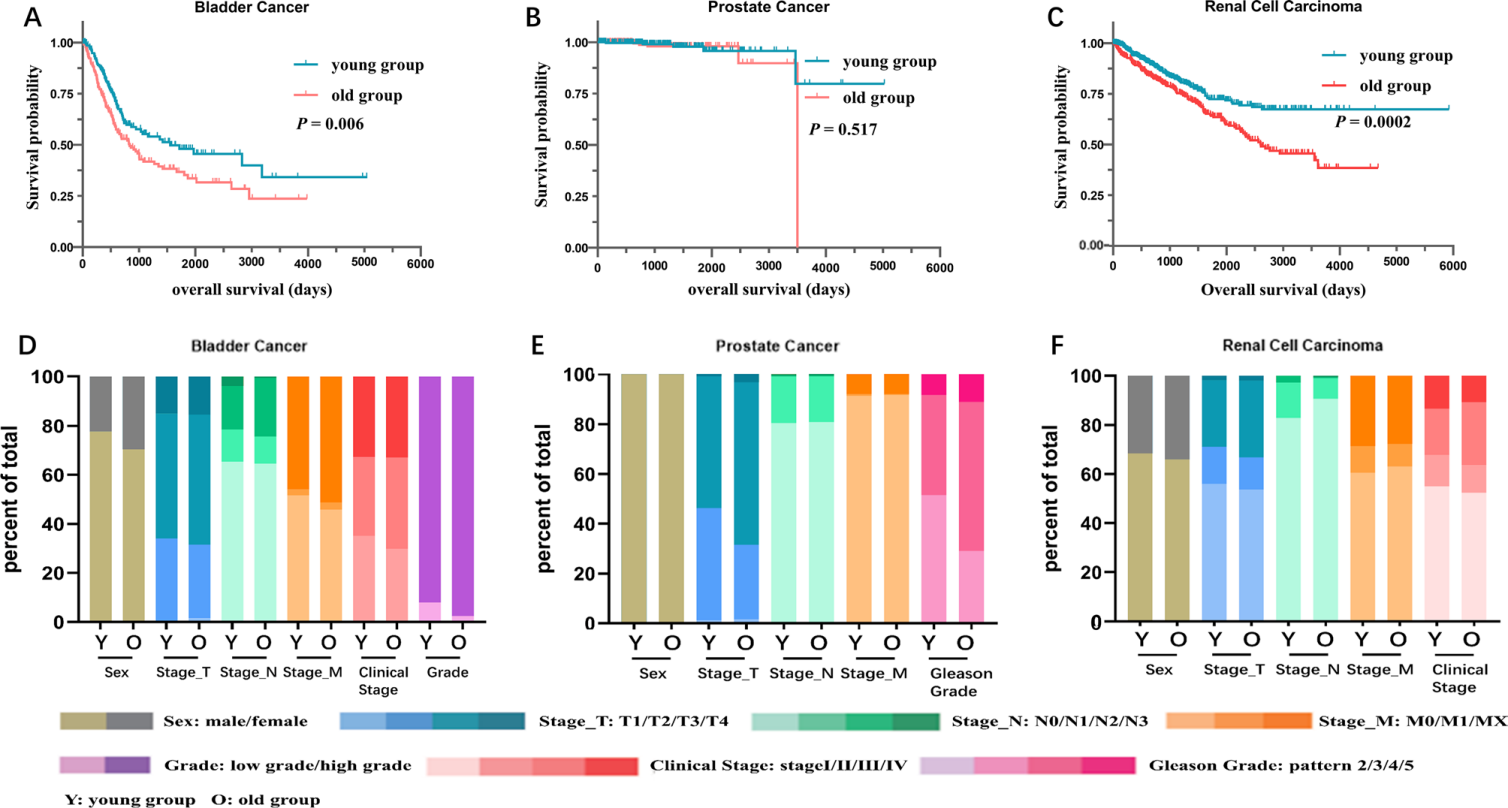
Effects of age on the clinical characteristics of urogenital cancers. (**A**–**C**) Survival rates of urogenital cancer patients in the old and young groups (red and blue lines, respectively). (**D**–**F**) Clinical characteristics of bladder cancer, prostate cancer, and renal cell carcinoma patients in the old and young groups. Columns represent groups, and colours represent clinical characteristics.

Kaplan–Meier analysis revealed that in bladder and kidney cancer, elderly patients had a significantly worse prognosis than younger patients; however, no significant difference in prognosis was observed between the two age groups among prostate cancer patients (Fig. 1A–C). Notably, only 10 of the 500 prostate cancer patients died, likely contributing to the lack of a significant difference in prognosis between the two groups. The proportion of T and M pathological stage patients was higher in the elderly group for all three cancer types, whereas no significant difference was observed in the proportions of young and elderly patients with N stage disease (Fig. 1D–F). High-grade bladder tumours and high Gleason stage prostate tumours were more frequent in elderly patients than in young patients.

### Identification of age-related DEGs

**Figure 2:**
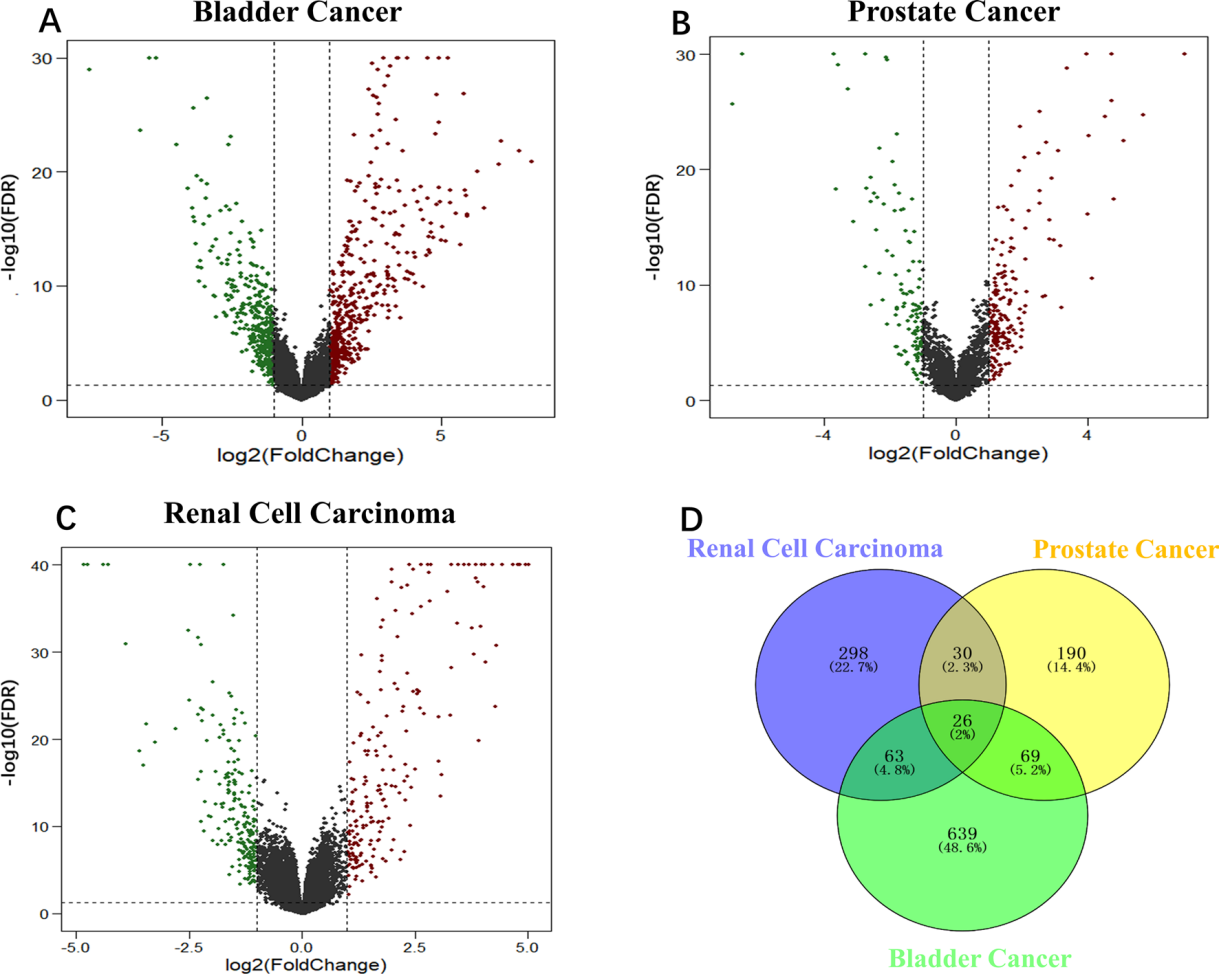
Identification of age-related DEGs. (**A**–**C**) Volcano plots showing the gene expression profiles of the different age groups in the three urogenital cancer types. Red/blue symbols indicate up-regulated/down-regulated genes with |log_2_ fold change (FC)| > 1 and adjust *P*-value < 0.05. (**D**) Venn diagram analysis (https://bioinfogp.cnb.csic.es/tools/venny/) was conducted to identify the common DEGs. We identified 188 genes that were differentially expressed in at least two cancer types.

The volcano map in Fig. 2A–C illustrates the distribution of DEGs in bladder, prostate, and renal cell cancers. The numbers of up-regulated/down-regulated DEGs were 453/344 for bladder cancer, 189/126 for prostate cancer, and 203/214 for renal cell carcinoma. All the DEGs table of kidney, prostate, bladder tumours with |log_2_ FC|, *P*-Value, etc. were shown in Tables S1–S3. Based on the findings of Venn diagram analysis (Fig. 2D), we selected 188 genes that were differentially expressed in at least two of the three cancer types. These common DEGs were used in the subsequent analysis.

### Enrichment analysis of the common DEGs

**Figure 3:**
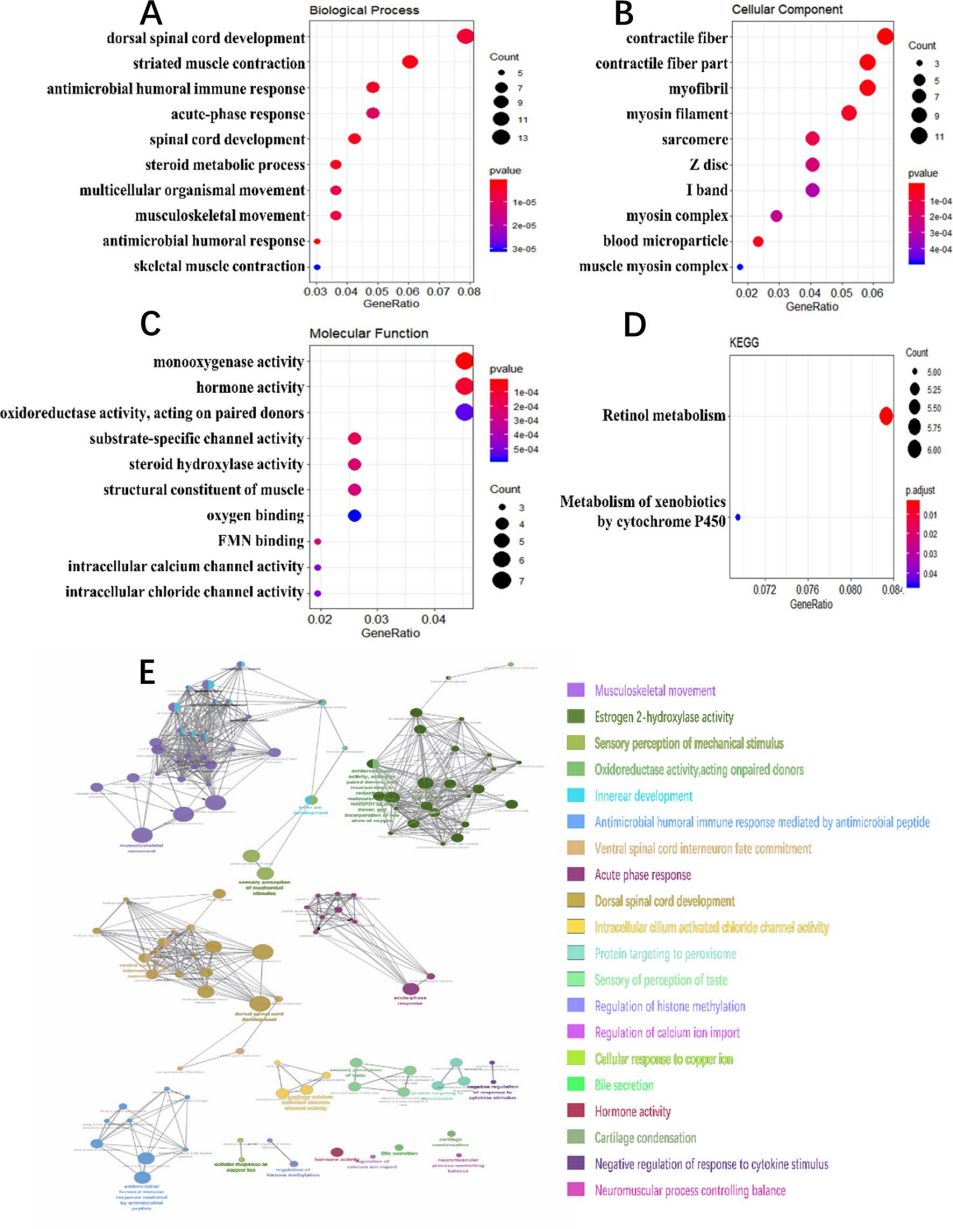
Enrichment analysis of the common DEGs. (**A**–**D**) Enrichment analyses of biological processes, cellular components, molecular functions, and KEGG pathways were conducted using clusterProfiler package of R software. The top 10 terms are shown. The colour indicates the enrichment significance (*P*-value) and the size indicates the number of genes. (**E**) Enrichment analysis of the common DEGs was also conducted using the clueGO Cytoscape plugin. Each point represents a gene, and different colours represent different enriched terms.

The clusterProfiler R package was used for functional enrichment analysis of the 188 common DEGs (Fig. 3A–D). GO enrichment analysis revealed that the most common biological processes among the DEGs were spinal cord development, striated muscle contraction, antimicrobial humoral response, immune response, and steroid metabolic process (Fig. 3A). Contractile fibre, myofibril, myosin filament, sarcomere, Z disc, I band, myosin complex, blood microparticle, and muscle myosin complex were the most significantly enriched cellular components (Fig. 3B). The most common molecular functions among the DEGs were monooxygenase activity, hormone activity, oxidoreductase activity, substrate-specific channel activity, steroid hydroxylase activity, structural constituent of muscle, oxygen binding, FMN binding, intracellular calcium channel activity, and intracellular chloride channel activity (Fig. 3C). KEGG pathway analysis revealed significant enrichment of the retinol and cytochrome P450-mediated metabolism of xenobiotic compounds (Fig. 3D).

Since there were only a few enriched pathways, we used clueGO to further analyse enriched pathways and biological processes. We identified musculoskeletal movement, sensory perception of mechanical stimuli, oxidoreductase activity, antimicrobial humoral immune response, ventral spinal cord interneuron fate commitment, acute phase response, dorsal spinal cord development, and intracellular cilium activated chloride channel activity as significantly enriched processes (Fig. 3E).

### PPI network and age-related hub genes

To investigate the interrelations of the 188 age-related DEGs and identify hub genes, we conducted a PPI analysis using different algorithms (MCC, DMNC, MNC, EPC, and degree). We identified top 30 hub genes of each algorithm, 14 of which were common among the five different PPI algorithms. The names and function of these 14 hub genes are shown in Table 1. These genes are differentially expressed in the TME of urinary tract cancer patients of different ages, likely contributing to the effects of age on the clinical characteristics of the patients.

**Table 1:** Functional roles of 14 hub genes.

No	Genes	Full name	Function
1	ORM1	Orosomucoid 1	Innate immune system and carvedilol pathway, pharmacokinetics
2	ORM2	Orosomucoid 2	Innate immune system and response to elevated platelet cytosolic Ca^2 +^
3	NEUROG3	Neurogenin 3	DNA-binding transcription factor activity and RNA polymerase II proximal promoter sequence-specific DNA binding
4	INSM1	Insulinoma-associated 1	Regulation of beta-cell development and developmental biology
5	MYBPH	Myosin binding protein H	G13 signaling pathway and actin nucleation by ARP-WASP complex
6	TRIM63	Tripartite Motif Containing 63	Ligase activity and obsolete signal transducer activity
7	MYLPF	Myosin, light chain 11, regulatory	Calcium ion binding and structural constituent of muscle
8	MYH6	Myosin heavy chain 6	Sertoli-sertoli cell junction dynamics and translocation of GLUT4 to the plasma membrane
9	CSRP3	Cysteine and glycine rich protein 3	Structural constituent of muscle and telethonin binding
10	MYH7	Myosin heavy chain 7	Sertoli-sertoli cell junction dynamics and translocation of GLUT4 to the plasma membrane
11	TCAP	Titin-Cap	Ion channel binding and structural constituent of muscle
12	TNNT3	Troponin T3, fast skeletal type	Actin binding and tropomyosin binding
13	MYH7B	Myosin heavy chain 7B	Myosin heavy chain 7B
14	HP	Haptoglobin	Serine-type endopeptidase activity and hemoglobin binding

### The relationship between the expression of hub genes and clinical characteristics

**Figure 4:**
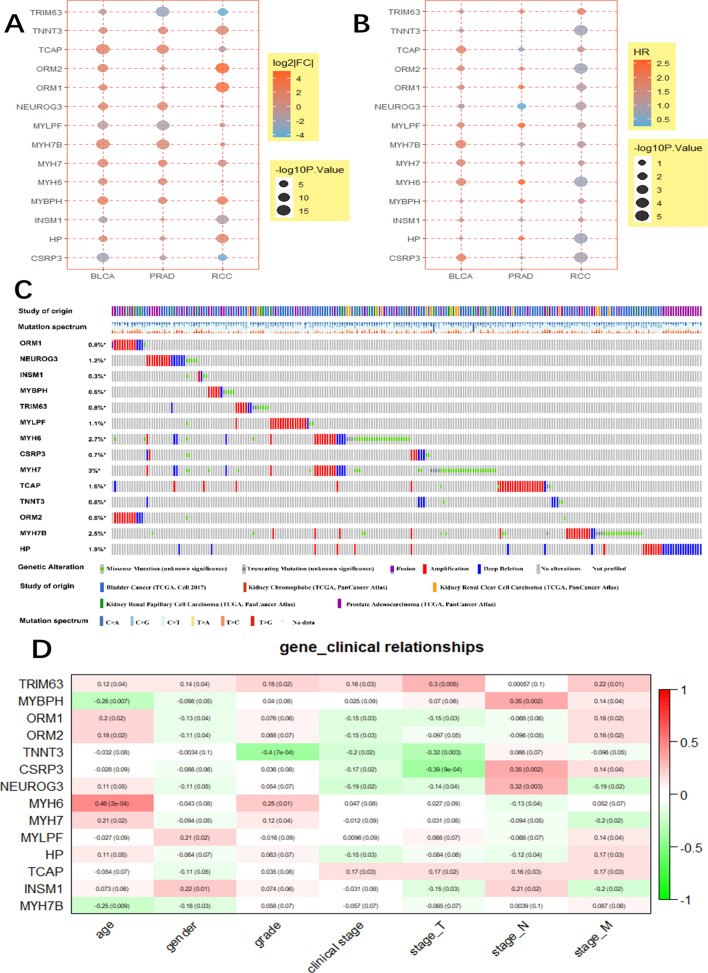
The relationship between the expression of hub genes and clinical characteristics. (**A**) mRNA levels of the 14 hub genes. The colour of each point represents the log_2_FC, and the size represents the *P*-value. (**B**) The association between the 14 hub genes and overall survival, as determined by univariate Cox regression analysis. The colour represents the hazard ratio (HR), and the size represents the *P*-value. (**C**) Summary of the mutations in the 14 hub genes in urogenital cancers via cBioPortal website (http://www.cbioportal.org/). Each row represents a gene, and each column represents a tumour sample. Red bars indicate gene amplifications, blue bars represent deep deletions, green squares indicate missense mutations, and grey bars indicate truncation mutations. (**D**) The relationship between the expression of the 14 hub genes and the clinical characteristics of bladder cancer. The numbers in rectangles indicate the correlation coefficient and the numbers in brackets indicate the *P*-value.

The mRNA levels of the 14 hub genes are shown in Fig. 4A, whereas Fig. 4C depicts the mutation frequency of hub genes. Elderly patients had a higher mutation frequency compared with young cancer patients. Notably, the mutation frequency of *MYH7*, *MHY6,* and *MYH7B* was considerably higher than that of the other hub genes, exceeding 2.5% (Fig. 4C). Meanwhile, ROC analysis results of these hub genes, namely Area Under Curve (AUC), are shown in Table 2, which showed significant differentiation in gene expression between the two groups.

**Table 2:** ROC analysis results of 14 hub genes.

Genes	Bladder cancer	Prostate cancer	Kidney cancer
ORM1	0.645	0.873	0.809
ORM2	0.831	0.915	0.663
NEUROG3	0.648	0.643	0.655
INSM1	0.663	0.832	0.662
MYBPH	0.636	0.656	0.666
TRIM63	0.874	0.841	0.833
MYLPF	0.816	0.656	0.651
MYH6	0.854	0.823	0.822
CSRP3	0.852	0.827	0.837
MYH7	0.626	0.815	0.663
TCAP	0.813	0.817	0.848
TNNT3	0.816	0.808	0.660
MYH7B	0.660	0.804	0.819
HP	0.887	0.814	0.815

We also assessed the relationship between the 14 hub genes and patient prognosis, and found that the prognostic value of the different hub genes varied according to cancer type (Fig. 4B). Importantly, *MYH6* expression levels were significantly associated with age in bladder cancer, while *MYBPH* exhibited a negative correlation with age (Fig. 4D).

### Co-expression analysis of hub genes

**Figure 5:**
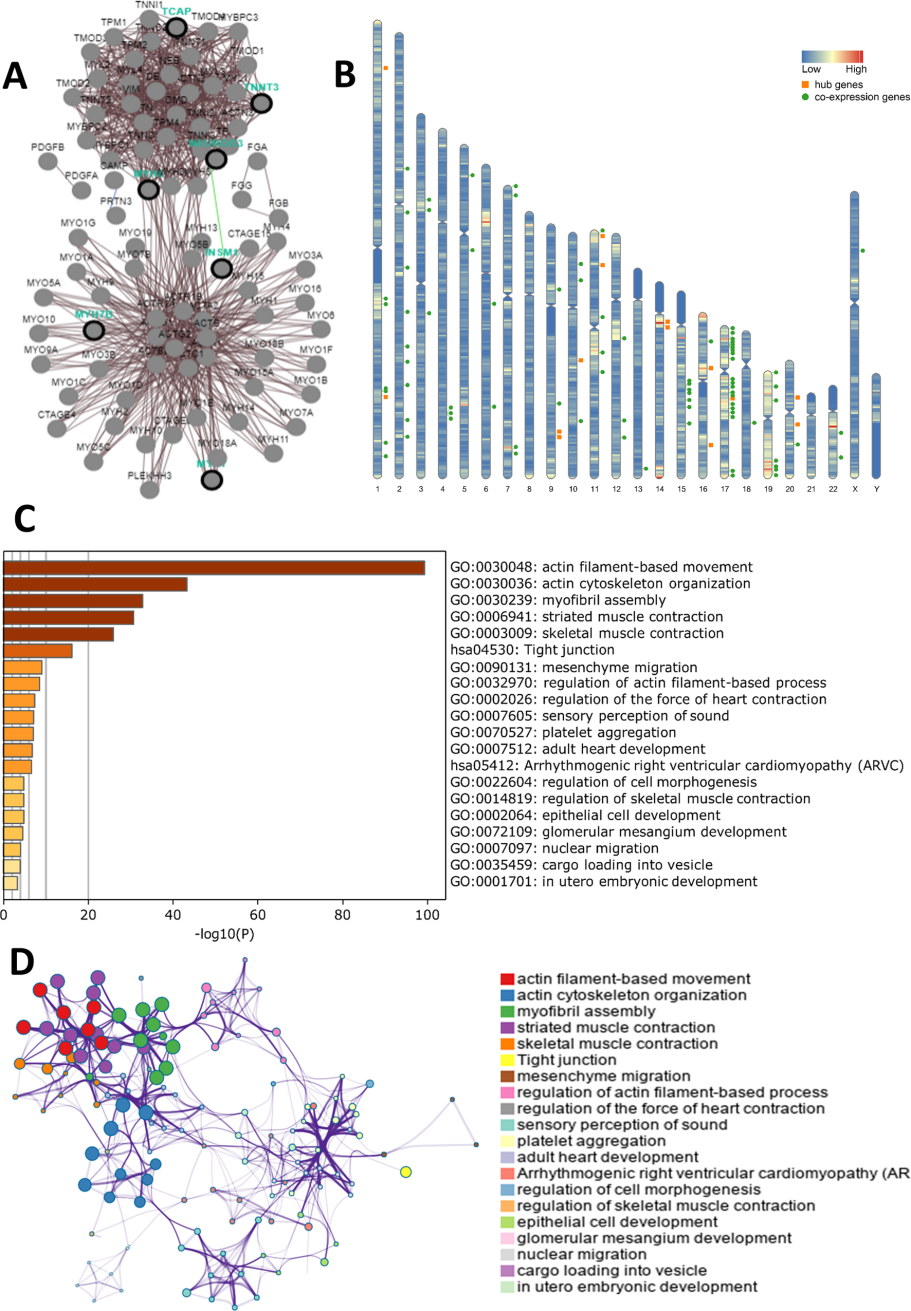
Co-expression analysis of the 14 hub genes. (**A**) The co-expression network was constructed using PCViz (http://www.pathwaycommons.org/pcviz). (**B**) Distribution of the 14 hub genes and 79 co-expressed genes on chromosomes. The orange squares represent hub genes, and the green circles represent co-expressed genes. (**C**) Bar graph of enriched terms among the hub and co-expressed genes, coloured based on the *P*-values. (**D**) The enrichment results via Metascape website (https://metascape.org/) were coloured according to cluster ID; nodes that share the same cluster ID are typically close to each other.

Co-expression analysis is often used to infer the function of poorly annotated genes. We identified 79 genes that were co-expressed with the 14 hub genes and constructed a PPI network (Fig. 5A). Here, genes with similar expressions and functions are closer to each other, thus forming a classic bicentric form of 'hairball'. The genes in the upper small hairball structure are mainly involved in the inflammation and immune response of the tissue, while the genes in the lower hairball are mainly involved in the composition and movement of muscle tissue. Chromosome distribution analysis of the co-expressed genes revealed that 21 of the 79 (22.5%) co-expressed genes were located on chromosome 17 (Fig. 5B). Hence, Chromosome 17 may have many age-related genes, which are involved in the regulation of age-related microenvironment changes and cancer initiation. Intriguingly, gene mutations on chromosome 17 have been associated with senile frontotemporal dementia and senile diseases, such as Parkinson’s disease^27^. Metascape analysis of the co-expressed genes revealed enrichment in muscle/ motor-related pathways, such as actin, myofibril, and skeletal muscle pathways, as well as in various cancer-related pathways, including interstitial migration and epithelial cell development (Fig. 5C,D). These pathways may be involved in the age-mediated epigenetic regulation of urogenital cancers.

### Age-related changes in cell infiltrates

**Figure 6:**
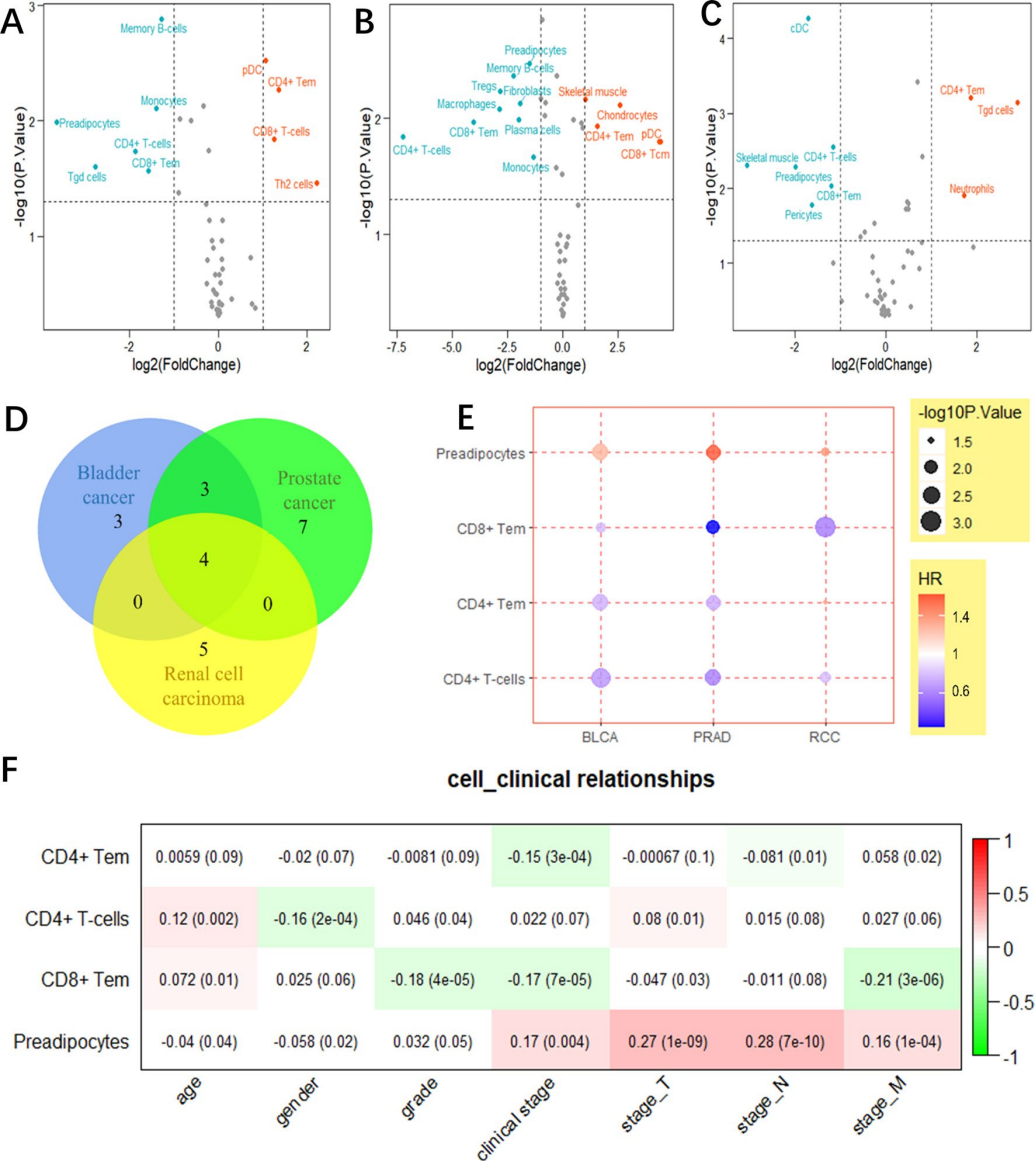
Age-related changes in cell infiltrates in the TME. (**A**–**C**) Volcano plots showing the levels of 48 cell types in the TME, as determined by the xCell method. The up-regulated cells in the old group are shown in red, and the down-regulated cells are shown in light blue. (**D**) Venn diagram showing the common age-related cell types among the three urogenital cancers. (**E**) The association between the four age-related cell types and overall survival, as determined by univariate Cox regression analysis. The colour represents the HR value, and the size represents the *P*-value. (**F**) The relationship between the levels of the four age-related cell types and the clinical characteristics of bladder cancer. The numbers in rectangles indicate the correlation coefficient and the numbers in brackets indicate the *P*-value.

Next, we analysed the levels of 48 cell types in the TME in old and young urogenital cancer patients (Fig. 6A–C). The levels of preadipocytes, CD8^+^ effector memory T (Tem), CD4^+^ T, and CD4^+^ Tem cells in the TME differed significantly between the two age groups in urogenital cancers (Fig. 6D). Preadipocytes levels were higher in the TME of elderly patients than young patients, whereas the levels of the other three immune cell types were lower. Interestingly, preadipocyte levels in the TME were associated with poor patient survival, whereas the levels of CD8^+^ Tem, CD4^+^ T, and CD4^+^ Tem cells were associated with improved survival (Fig. 6E). Consistently, preadipocyte levels in the TME were strongly correlated with the clinical TNM stage in bladder cancer patients (Fig. 6F).

### The relationship between age-related cells and hub genes

**Figure 7:**
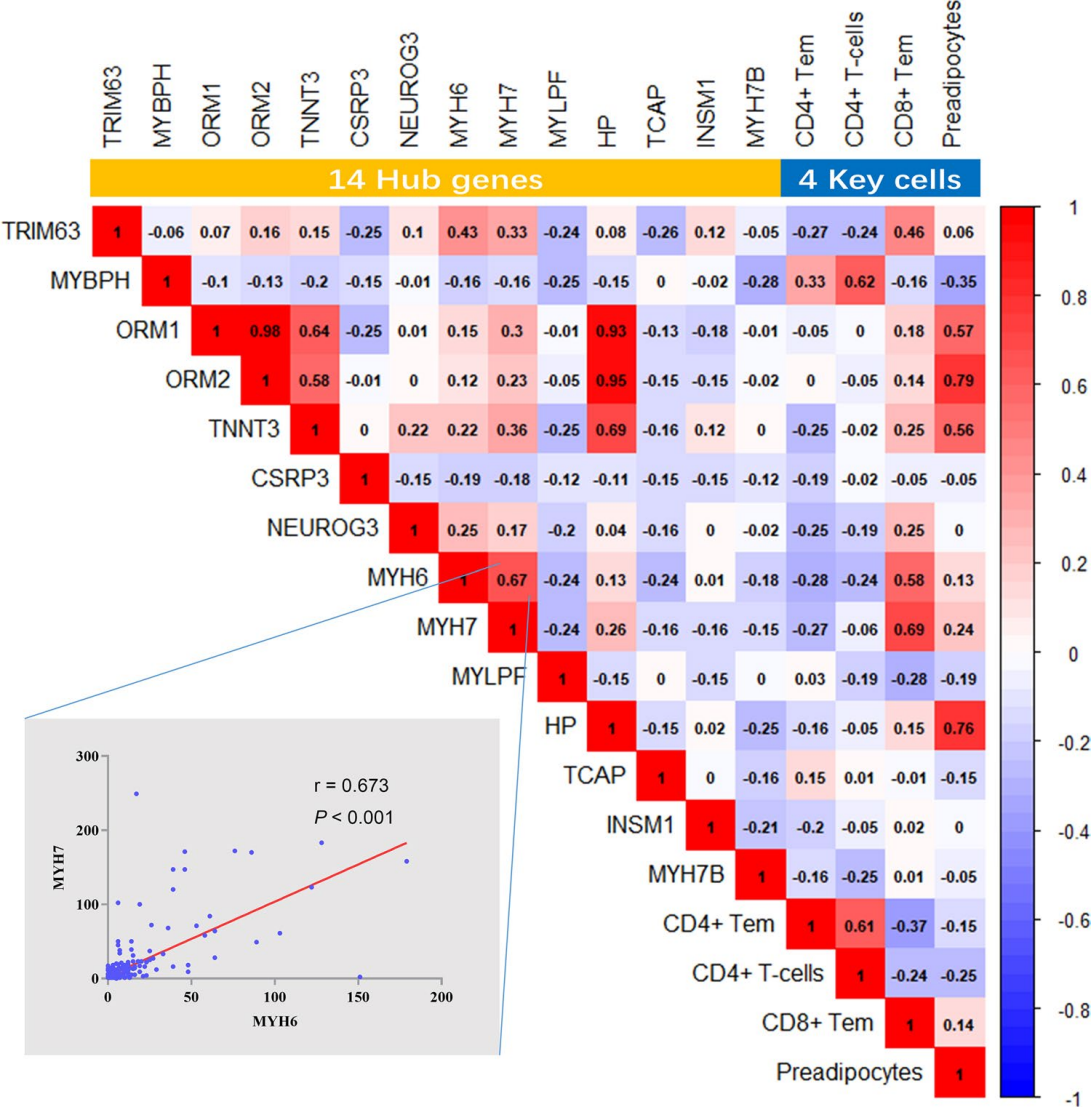
The relationship between age-related cell types and hub genes in bladder cancer. Correlation analysis of age-related cells and hub genes in bladder cancer patients and the figure was made via ggplot R package; the colours of the squares represent the correlation coefficient (Pearson correlation). The correlation map at the bottom left shows the correlation between *MYH6* and *MYH7*.

Correlation analysis of age-related cells and hub genes revealed a strong association between preadipocyte levels in the TME and the expression levels of *ORM1*, *ORM2* and *HP* (Fig. 7). Additionally, there was a strong positive correlation between the levels of CD4^+^ T and CD4^+^ Tem cells in the TME.

## Discussion

The incidence of kidney, prostate, and bladder cancer increases with age, and 82% of newly diagnosed urogenital cancer patients are aged 60 years and older^28^. Although the strong links between ageing and tumorigenesis have become evident, the age-related changes in the TME in urogenital cancer patients remain elusive. Advances in sequencing technologies and bioinformatics tools have enabled unbiased characterisation of age-related changes in the microenvironment in urogenital cancer. In this study, we identified 14 hub genes and 4 cell types enriched in the ageing TME. The genes and cell types identified in this study may mediate age-induced genomic and clinicopathological alterations in urogenital cancers.

Age is one of the major factors affecting the occurrence of patients with urogenital cancers. In this study, we found that elderly patients with bladder or renal cell cancer had poorer overall survival and a higher clinical stage than younger patients, consistent with previous findings^29,30^. However, we found no significant difference in prognosis between the two age groups in prostate cancer. It is worth noting that only 10 of 500 prostate cancer patients had died, making it statistically challenging to assess differences in patient prognosis. A study of 259 prostate cancer patients showed that older patients had poorer cancer-specific survival than younger patients^31^.

In this study, we identified 14 hub genes associated with age-related changes in the TME (Table 1), which may contribute to differences in the overall survival and clinicopathological characteristics of patients in different age groups. ORM1 and ORM2 encode endoplasmic reticulum membrane proteins regulating lipid homeostasis and protein quality control^32^. HP expression levels were strongly associated with those of ORM1 and ORM2. Interestingly, all three genes have been implicated in various inflammatory diseases, such as sarcoidosis and chronic obstructive pulmonary disease^33,34^. MYH6, MYH7, and MYH7B are myosin heavy chain proteins, while MYLPF is a myosin light chain protein and MYBPH a myosin-binding protein. Mutations in these proteins have been linked to various muscle-related disorders and heart diseases^35^. CSRP3 is an autophagy-regulating protein essential for degrading muscle-related components and maintaining muscle structure and function^36^. TCAP is a giant elastic protein with kinase activity that extends half the length of a sarcomere and maintains cellular structure^37^. TNNT3 is a fast skeletal muscle troponin T (TnT) initiating muscle contractions^38^. TRIM63 regulates proteasome degradation of cardiac troponin I/TNNI3 and other sarcomeric-associated proteins^39^. INSM1 and NEUROG3 are essential markers for pancreatic neuroendocrine tumours and have been implicated in gastrointestinal tumours, diarrhoea, and malabsorption^40,41^. Hence, these hub genes are mainly involved in muscle structure, metabolism, and inflammation/immune. Interestingly, premature aging and muscle tissue expression disorders were often detected in immunodeficiency mice, and MYH7 and TRIM63 gene expression disorders were also detected^42,43^. In addition, HP and ORM1 were related to immune response, and the expression was decreased in immunodeficiency virus (HIV) infected patients^44,45^. These evidences seem to suggest a link between muscle tissue disorders/immunodeficiency and ageing/cancer. Their relationship with ageing and carcinogenesis required further investigation.

Enrichment analysis of genes co-expressed with the 14 hub genes revealed their role in muscle structure and contraction regulation, ion channel (Cl^−^, Ca^2+^) regulation, inflammation, antibacterial humoral immune responses, and metabolism, in addition to various cancer-related pathways. Cancer is widely considered as an age-related disease. Cancer and ageing share various biological processes, including changes in intracellular communication, protein stability, and metabolism, as well as mitochondrial dysfunction^46^. In this study, we found that changes in muscle activity and the immune system were common to both ageing and cancer. Myocardial and skeletal muscle motor capabilities decrease with ageing, and the accumulation of mutations can cause organ dysfunction in older individuals^47^. Furthermore, both cancer and ageing are characterised by disruption of metabolic homeostasis, including enhanced glycolysis and impaired oxidative phosphorylation^48,49^. Changes in the levels of steroids, including adrenocorticosteroid, androgen, and oestrogen, can promote cancer development and progression^50^. Disruption of immune homeostasis is also shared by ageing and cancer, and numerous studies have linked immunosenescence to cancer^13,14^. However, more studies are required to elucidate the mechanistic links between cancer and ageing.

In this study, we identified preadipocytes, CD4^+^ Tem cells, CD4^+^ T cells, and CD8^+^ Tem cells as cell markers of age-related changes in the TME. Ageing significantly affects the TME, promoting tumour progression and metastasis^13^. Interestingly, the levels of tumour-infiltrating fibroblasts in elderly prostate patients were found to be particularly low. Preadipocytes levels were strikingly high in the TME of elderly patients. The increase in adipose tissues is one of the hallmarks of ageing, likely reflecting a link between ageing and cancer^46,51^. Preadipocytes are closely related to macrophages and are often dedifferentiated during ageing, switching to a pro-inflammatory, tissue-remodelling, senescent-like state^51^. A recent study has shown that preadipocyte dedifferentiation is regulated by the JAK pathway^52^. The levels of tumour-infiltrating CD4^+^ Tem cells, CD4^+^ T cells, and CD8^+^ Tem cells were lower in our elderly patients than in the young patients. Immune dysfunction is a common feature of ageing; this phenomenon is known as immunosenescence and has been linked to impaired immune surveillance and increased risk of cancer development^14^. Immunosenescence is associated with extensive alterations in the immune system, and CD4^+^ and CD8^+^ T cells are particularly susceptible to immunosenescence^53^. Meanwhile, immunodeficiency and immunosenescence are both manifested as decreased or absent immune cell content. CD4+ T cells and CD8+ T cells were significantly reduced in common immunodeficiency diseases^54^. The incidence of multiple cancers is increased in patients with acquired immune deficiency, which also reflects the influence of immune deficiency on the promotion of tumour incidence to some extent^55^. In addition, patients with immunodeficiency are more likely to develop flora disorder and urinary tract infections, which may also increase the incidence of corresponding tumours^56,57^. Therefore, the infiltration level of immune cells is related to the tumorigenesis, and modulating the infiltration of these four cells in TME may provide a novel platform to treat cancer.

This study is not without limitations. First of all, this study was only analysed in urogenital cancers and may not be applicable to all tumours. Secondly, matched adjacent-normal expression data is very rarely available in tumour datasets so this is probably just a fact of life for this kind of study but it does mean that it is not possible to correct for general effects of aging unrelated to cancer. It is notable for example that most of the DEGs identified were not obviously related to a possible age-related tumour response e.g. immunosenesence, but rather to pathways one might expect to be affected by normal aging processes e.g. muscle. Additionally, muscle tissue changes and immunosenesence, as common features of elderly patients, may be associated with tumorigenesis. However, whether muscle tissue changes and immunosenesence are a cause or an effect of tumorigenesis need a further study.

In conclusion, we identified 14 genes and 4 cell types associated with age-related changes in the TME in urogenital cancer patients. These genes and cell types may play an important role in the age-related differences in the clinicopathological characteristics of urogenital cancer patients, thus linking ageing with cancer development and progression. Further studies are required to determine the clinical relevance of these age-related genes and cell types to treatments of cancer and other age-related diseases tailored based on age.

## Supplementary Information

Supplementary Table S1.

Supplementary Table S2.

Supplementary Table S3.

Supplementary legends.

## Data Availability

Publicly available datasets were analyzed in this study, these can be found in the Cancer Genome Atlas (https://portal.gdc.cancer.gov/).
