# Supplementary material for: Transcriptomics analysis for the identification of potential age-related genes and cells associated with three major urogenital cancers
Source: Sci Rep. 2021 Jan 12;11:641. doi: 10.1038/s41598-020-80065-y (PMC7803945; doi:10.1038/s41598-020-80065-y)
Supplement: Supplementary file 4 — Supplementary legends. [file 41598_2020_80065_MOESM4_ESM.docx]

Transcriptomics analysis for the Identification of Potential Age-related Genes and Cells Associated with Three Major Urogenital Cancers

Jinlong Cao^1, 2^, Jianpeng Li^1, 2^, Xin Yang^3^, Pan Li^1, 2^, Zhiqiang Yao^1, 2^, Dali Han^1, 2^, Lijun Ying^1, 2^, Lijie Wang^4^, Junqiang Tian^1, 2, *^

^1^ Department of Urology, the Second Hospital of Lanzhou University, Lanzhou, 730000, People’s Republic of China,

^2^ Key Laboratory of Urological Diseases of Gansu provincial, Lanzhou 730000, People’s Republic of China,

^3^ Reproductive Medicine Center, the Second Hospital of Lanzhou University, Lanzhou, 730000, People’s Republic of China,

^4^ Department of Gynecology, the Second Hospital of Lanzhou University, Lanzhou, 730000, People’s Republic of China

***** corresponding. Junqiang Tian. E-mail: ery___tianjq@lzu.edu.cn

Jinlong Cao, Jianpeng Li, Xin Yang contributed equally to this work.

**Supplementary Materials**

Table S1: Differentially expressed genes of kidney cancer. Table S2: Differentially expressed genes of prostate cancer. Table S3: Differentially expressed genes of bladder cancer.
